# Silencing of *Abcc8* or inhibition of newly upregulated Sur1-Trpm4 reduce inflammation and disease progression in experimental autoimmune encephalomyelitis

**DOI:** 10.1186/s12974-015-0432-3

**Published:** 2015-11-18

**Authors:** Tapas K. Makar, Volodymyr Gerzanich, Vamshi K.C. Nimmagadda, Rupal Jain, Kristal Lam, Fahad Mubariz, David Trisler, Svetlana Ivanova, Seung Kyoon Woo, Min Seong Kwon, Joseph Bryan, Christopher T. Bever, J. Marc Simard

**Affiliations:** Research Service and MS Center of Excellence, Veterans Affairs Maryland Health Care System, Baltimore, MD 21201 USA; Department of Neurology, University of Maryland School of Medicine, Baltimore, MD 21201 USA; Department of Neurosurgery, University of Maryland School of Medicine, Baltimore, MD 21201 USA; Department of Pathology, University of Maryland School of Medicine, Baltimore, MD 21201 USA; Department of Physiology, University of Maryland School of Medicine, Baltimore, MD 21201 USA; Neurosurgical Service, Veterans Affairs Maryland Health Care System, Baltimore, MD 21201 USA; Pacific Northwest Diabetes Research Institute, 720 Broadway, Seattle, WA 98122 USA; Department of Neurosurgery, 22 S. Greene St., Suite S12D, Baltimore, MD 21201-1595 USA

**Keywords:** Multiple sclerosis, Experimental autoimmune encephalomyelitis, Sur1, Trpm4, Sur1-Trpm4 channel, *Abcc8*, Glibenclamide, Astrocyte, Inflammation, Demyelination and neuroprotection

## Abstract

**Background:**

In experimental autoimmune encephalomyelitis (EAE), deletion of transient receptor potential melastatin 4 (*Trpm4*) and administration of glibenclamide were found to ameliorate disease progression, prompting speculation that glibenclamide acts by directly inhibiting Trpm4. We hypothesized that in EAE, Trpm4 upregulation is accompanied by upregulation of sulfonylurea receptor 1 (Sur1) to form Sur1-Trpm4 channels, which are highly sensitive to glibenclamide, and that Sur1-Trpm4 channels are required for EAE progression.

**Methods:**

EAE was induced in wild-type (WT) and *Abcc8*−/− mice using myelin oligodendrocyte glycoprotein 35–55 (MOG_35–55_). Lumbar spinal cords were examined by immunohistochemistry, immuno-Förster resonance energy transfer (immunoFRET), and co-immunoprecipitation for Sur1-Trpm4. WT/EAE mice were administered with the Sur1 inhibitor, glibenclamide, beginning on post-induction day 10. Mice were evaluated for clinical function, inflammatory cells and cytokines, axonal preservation, and white matter damage.

**Results:**

Sur1-Trpm4 channels were upregulated in EAE, predominantly in astrocytes. The clinical course and severity of EAE were significantly ameliorated in glibenclamide-treated WT/EAE and in *Abcc8*−/−/EAE mice. At 30 days, the lumbar spinal cords of glibenclamide-treated WT/EAE and *Abcc8*−/−/EAE mice showed significantly fewer invading immune cells, including leukocytes (CD45), T cells (CD3), B cells (CD20) and macrophages/microglia (CD11b), and fewer cells expressing pro-inflammatory cytokines (TNF-α, IFN-γ, IL-17). In both glibenclamide-treated WT/EAE and *Abcc8*−/−/EAE mice, the reduced inflammatory burden correlated with better preservation of myelin, better preservation of axons, and more numerous mature and precursor oligodendrocytes.

**Conclusions:**

Sur-Trpm4 channels are newly upregulated in EAE and may represent a novel target for disease-modifying therapy in multiple sclerosis.

## Background

Multiple sclerosis (MS) is an autoimmune disease marked by chronic inflammation, demyelination, and neurodegeneration of the central nervous system (CNS) that causes neurological disability in young and middle-aged adults [1, 2]. MS has no known cure, and patients suffer from progressive disability due to irreversible neurological damage. MS and its principal animal model, experimental autoimmune encephalomyelitis (EAE), are characterized by myelin-specific autoreactive T cells that enter the CNS and initiate inflammation and tissue damage leading to oligodendrocyte cell death, axonal demyelination, and neuronal degeneration [3]. Inflammation is perpetuated by both infiltrating immune cells and by astrocytes [4–9]. Of the currently approved disease-modifying therapies for MS, most target immune cells or a pro-inflammatory cytokine [10], with the only exception being fingolimod (FTY720), which has effects directly on astrocytes [11, 12].

Emerging evidence indicates that transient receptor potential melastatin 4 (Trpm4) plays a crucial role in the pathophysiology of various CNS injuries. When upregulated and activated, Trpm4 contributes to the formation of cytotoxic edema, and it functions as an end-executioner in accidental necrotic death induced by Ca^2+^ overload, adenosine triphosphate (ATP) depletion, or reactive oxygen species [13, 14]. Trpm4 is upregulated in microvascular endothelial cells, neurons, and glial cells in preclinical rat models of stroke, spinal cord injury, and subarachnoid hemorrhage [15–19].

Recently, Schattling et al. [20] implicated Trpm4 upregulation in the pathophysiology of EAE and showed that *Trpm4* deletion was associated with reduced disease severity and improved recovery following EAE induction. They also showed that glibenclamide ameliorates clinical signs of EAE, and they speculated that the salutary effects of glibenclamide were due to direct blockade of Trpm4 [20]. However, given the low potency of glibenclamide inhibition of Trpm4 [17], a direct effect of glibenclamide on Trpm4 seems unlikely.

Sulfonylurea receptor 1 (Sur1) is an ATP-binding cassette transporter family member that functions as a regulatory subunit when it co-assembles with heterologous pore-forming subunits to form cation channels. The most widely recognized association is with the ATP-sensitive K^+^ channel, Kir6.2, with which it forms Sur1-Kir6.2 (K_ATP_) channels that are constitutively expressed in pancreatic β cells and are linked to diabetes mellitus [21–23]. Sur1 also associates with Trpm4 to form Sur1-Trpm4 channels that are transcriptionally upregulated in the brain and spinal cord following ischemic, traumatic, and inflammatory CNS injuries [17–19]. A crucial property of the Sur1-Trpm4 channel is that both subunits, Sur1 and Trpm4, must be upregulated and functional for the manifestation of its pathological effects, with deletion or pharmacological blockade of either subunit resulting in equivalent abrogation of injury severity [24].

We hypothesized that in EAE, Trpm4 upregulation, as reported by Schattling et al. [20], is accompanied by upregulation of Sur1, that the two proteins co-assemble to form Sur1-Trpm4 channels (which are highly sensitive to glibenclamide [17]), and that Sur1-Trpm4 channels, rather than Trpm4 channels alone, are required for disease progression and for manifestation of glibenclamide sensitivity in EAE. Here, we assessed this hypothesis in a murine EAE model using gene silencing and pharmacological inhibition of Sur1.

## Methods

### Murine EAE model

All experiments were conducted in accordance with the guidelines of the National Institutes of Health and under a protocol approved by the Institutional Animal Care and Use Committee of the University of Maryland School of Medicine. Female C57BL/6 J mice were obtained from The Jackson Laboratory (Bar Harbor, ME). *Abcc8−/−* mice, obtained as described [25], exhibited neurological function, gait, and spinal cord histology indistinguishable from WT. Mice were housed under pathogen-free conditions in the animal facility of the University of Maryland School of Medicine.

EAE was induced in female WT and *Abcc8*−/− mice at 8 weeks of age, as described [26, 27]. EAE was induced with MOG_35–55_ peptide (Biomer Technology, Pleasanton, CA, USA) in complete Freund’s adjuvant (Sigma-Aldrich, St Louis, MO) containing *Mycobacterium tuberculosis* (H37RA, Difco Laboratories, Detroit, MI). Mice were immunized by subcutaneous injection in the flank regions (left and right sides) with 200 μL total of an emulsion of MOG_35–55_ peptide (200 μg in 100 μL PBS plus 100 μL of complete Freund’s adjuvant containing 0.4 mg of heat-inactivated *M. tuberculosis*). Each mouse then received 400 ng of pertussis toxin (List Biological Laboratories) intraperitoneally (IP) on post-induction day (pid) 0 and pid-2.

### Glibenclamide treatment

After the onset of clinical symptoms (>20 % of WT/EAE mice with clinical scores of 1 or greater; pid-10), 10 μg glibenclamide was administered daily by IP injection to WT/EAE mice in the treatment group until the end of the experiment (pid-30). A stock solution of glibenclamide was prepared by placing 25 mg of glibenclamide (#G2539; meets USP testing; Sigma, St. Louis, MO) into 10 mL dimethyl sulfoxide (DMSO). We diluted 200 μL of this solution in 9.8 mL PBS; mice received 100 μL of this solution.

### Clinical evaluation

Scoring of disease severity was carried out as described [26, 27]. From pid-0 onwards, mice were assessed daily for signs of paralysis by two independent observers in a blinded fashion. Mice were assigned a clinical score of increasing severity: 1, limp tail; 2, hind limb paresis; 3, complete hind limb paralysis; 4, hind limb paralysis and body/front limb paresis/paralysis; 5, moribund. End point evaluation included mean severity of disease over time and mean day of disease onset (first day of score >0). Paralyzed mice (scores 3 and 4) were moved to individual cages where food and water were placed at cage floor level. The weight of EAE mice was measured every 2 days and mice were euthanized if there was a loss of more than 20 % in weight or if they become dehydrated.

### Histology, immunochemistry, and immunoFRET

On pid-10 or pid-30, mice were euthanized and transcardially perfused with NS (10 ml) followed by 10 % neutral buffered formalin (15 ml). The spinal cords were removed, and 7 μm cryosections or paraffin sections were prepared from the lumbar region.

Fluorescence immunohistochemistry and immuno-Förster resonance energy transfer (immunoFRET) were performed on cryosections from pid-10 and pid-30 using custom anti-Sur1 and anti-Trpm4 antibodies, as described [17]. Controls for immunoFRET included omission of one of the two primary antibodies. Co-localization analysis was performed using the algorithm in Nikon NIS imaging software, based on regions of interest (400 × 200 μm) positioned in white matter. Specific signals were defined as fluorescence intensity twice that of background. Co-localization of fluorescence signals in double immunolabeled sections was computed as Pearson’s correlation coefficient [28].

Paraffin sections from pid-30 mice were stained with hematoxylin and eosin (H&E) (for inflammatory cell infiltrates) or Luxol fast blue (LFB) (for demyelination) following standard protocols. Axonal loss was determined by silver nitrate (AgNO_3_) staining using Hito Bielschowsky OptimStain Kit (#HTKNS1126, Hitobiotec Inc., Wilmington, DE, USA). Slides were examined using bright-field microscopy.

Chromagen immunohistochemistry was performed on paraffin sections from pid-30, as described [29, 30], using VECTASTAIN Elite ABC Kits (#PK-6100) and Mouse on Mouse (M.O.M.) Elite Peroxidase Kit (#PK-2200) (Vector Laboratories, Burlingame, CA). Primary antibodies were directed against the following: CD45 (1:1500; #ab10558; Abcam, Cambridge, MA); CD3 (1:200; #ab5690; Abcam); CD20 (1:100; #sc-7735; Santa Cruz Biotechnology, Santa Cruz, CA); CD11b (1:800; #NB110-89474; Novus Biologicals, Littleton, CO); TNF-α (1:500; #sc-1350; Santa Cruz Biotechnology); IFN-γ (1:100; #bs-0480R; Bioss, Woburn, MA); IL-17 (1:50; #sc-7927; Santa Cruz Biotechnology); IL-10 (1:50; #sc-1783; Santa Cruz Biotechnology); MBP (1:500; #ab40390; Abcam); CNPase (1:1000; #MAB326; EMD Millipore, Billerica, MA); Olig-2 (1:2000; #MABN50; EMD Millipore); PDGFR-α (1:500; #sc-338; Santa Cruz Biotechnology); and SMI-312 (1:1000; #SMI-312R; Covance Inc., Gaithersburg, MD). Nuclei were counterstained with hematoxylin. The specificity of the immunostaining for all proteins was tested in control slides by incubation with pre-immune serum or after pre-adsorption of the antibody with the respective peptides used as immunogens. Slides were examined using bright-field microscopy.

Quantification of tissue stains and of chromagen immunolabelings was performed by blinded observers using Image J software (NIH, USA). Tissue stains and markers (H&E, LFB, MBP, CNPase, SMI312, AgNO3) were quantified by counting the number of positive/negative quadrants and expressing the percentage over the total number of quadrants examined. All cell labeling experiments were quantified based on an analysis of 8–10 fields per section, randomly positioned in white matter (CD45, CD3, CD20, CD11b, TNF-α, IFN-γ, IL-17, and Olig-2) or in tissues surrounding the central canal (PDGFR-α), with each field being 435 × 325 μm.

### Co-immunoprecipitation

Immunoblot and co-immunoprecipitation were performed on lumbar spinal cord tissues from pid-30 using custom anti-Sur1 and anti-Trpm4 antibodies, as described [17].

### Statistical analysis

Statistical analyses were performed using Prism software (GraphPad, San Diego, CA). Data are shown as mean ± SEM. Statistical significance between groups was determined using one-way analysis of variance (one-way ANOVA) with Fisher’s post-hoc comparisons. In all experiments, *P* < 0.05 was considered to be statistically significant.

## Results

### Sur1-Trpm4 heteromers in EAE mice

EAE was induced in WT and *Abcc8*−/− mice using MOG_35–55_. Tissues from the lumbar region of the spinal cord, where the disease is most prominent [31], were examined at pid-10 and pid-30. Tissues were immunolabeled for Sur1, Trpm4, and cell-specific markers.

**Fig. 1:**
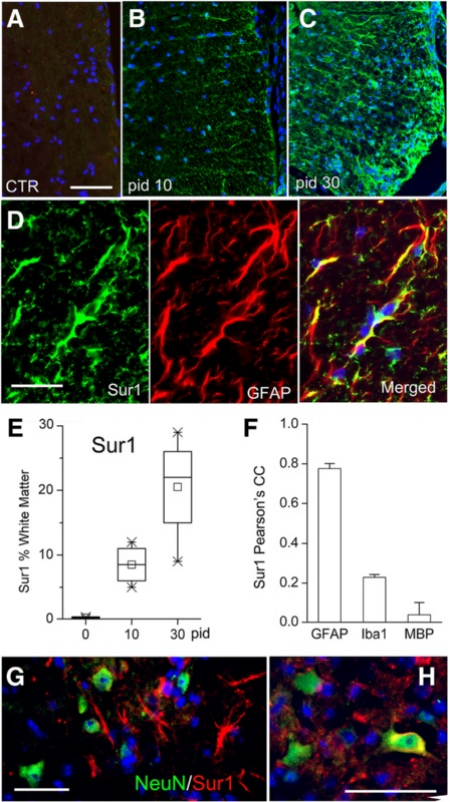
Upregulation of Sur1 in astrocytes in EAE. **a**–**c** White matter of lumbar spinal cord sections from control (CTR) (**a**) or WT/EAE mice on pid-10 or pid-30, as indicated (**b**, **c**), immunolabeled for Sur1; all imaging parameters were identical for the three panels; nuclei stained with DAPI. **d** Lumbar spinal cord section from a pid-30 WT/EAE mouse immunolabeled for Sur1 (*green*; *left panel*) and co-labeled for GFAP (*red*; *middle panel*) to identify astrocytes; merged images are also shown (**d**, *right panel*). **e** Box plots showing the percent of white matter with Sur1 immunopositivity under control conditions (pid-0) and at pid-10 and pid-30; five mice/group. **f** Bar graph showing Pearson’s correlation coefficient for Sur1 co-localization with GFAP, Iba1, and MBP; five mice/group. **g**, **h** Gray matter of lumbar spinal cord section from a pid-30 WT/EAE mouse double-labeled for Sur1 (*red*) and co-labeled for NeuN (*green*) to identify neurons; merged images are shown. The results illustrated are representative of findings in five mice/group; scale bars, 100 μm (**a**–**c**); 50 μm (**d**, **g**, **h**)

In white matter, Sur1 was not detected in normal WT controls (without EAE induction) (Fig. 1a). Modest Sur1 immunolabeling was present in the white matter of WT/EAE mice on pid-10, whereas by pid-30, Sur1 immunolabeling in white matter was widespread and robust (Fig. 1b, c). Quantitative analysis of Sur1 immunopositivity confirmed a progressive increase in white matter over the course of 30 days post-immunization (Fig. 1e). High power views showed that Sur1 expression was localized predominantly to cells with a stellate morphology, consistent with astrocytes (Fig. 1d). Double immunolabeling confirmed that Sur1 expression was localized predominantly to GFAP^+^ astrocytes (Fig. 1d). Quantitative analysis of the double immunolabeling data revealed a high degree of co-localization of Sur1 with GFAP (Pearson’s correlation coefficient (PCC), 0.78) and minimal co-localization of Sur1 with microglial Iba1 (PCC, 0.22) and oligodendrocyte MBP (PCC, 0.04) (Fig. 1f), consistent with most of the newly upregulated Sur1 being localized to astrocytes.

In gray matter, as in white matter, most of the Sur1 expressed by pid-30 was identified in stellate cells, consistent with astrocytes, and most NeuN^+^ neurons showed little or no detectable Sur1 (Fig. 1g). Rarely, a “basket” of Sur1 immunopositivity was seen surrounding a neuron (Fig. 1h).

**Fig. 2:**
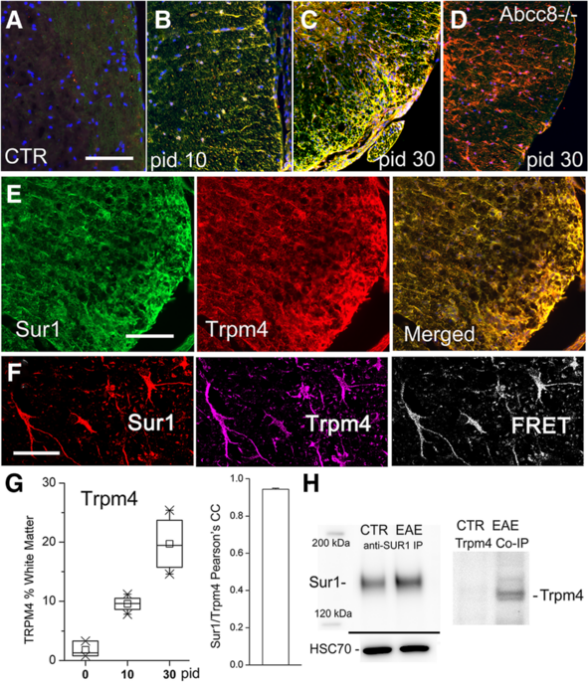
Upregulation of Sur1-Trpm4 channels in astrocytes in EAE. **a**–**e** White matter of lumbar spinal cord sections from control (CTR) (**a**), pid-10 (**b**), pid-30 (**c**–**e**) WT/EAE (**b**, **c**, **e**), and *Abcc8*−/−/EAE (**d**) mice, immunolabeled for Sur1 and co-labeled for Trpm4; merged images are shown in (**a**–**d** and **e**, *right panel*), demonstrating extensive co-localization (*yellow*) of Sur1 and Trpm4; secondary antibodies were conjugated with Alexa Fluoro 488 (*green*) or Cy3 (*red*). **f** Lumbar spinal cord section from a pid-30 WT/EAE mouse, immunolabeled for Sur1 (*left panel*), and co-labeled for Trpm4 (*middle panel*); immunoFRET imaging is also shown (*right panel*), demonstrating co-assembly of Sur1 and Trpm4; secondary antibodies were conjugated with Cy3 (*red*) or Cy5 (*purple*). **g** Box plots (*left panel*) showing the percent of white matter with Trpm4 immunopositivity under control conditions (pid-0) and at pid-10 and pid-30; bar graph (*right panel*) showing Pearson’s correlation coefficient for Sur1 and Trpm4 co-localization; five mice/group. **h** Immunoblot (*left panel*) showing upregulation of Sur1 in WT/EAE compared to control; HSC-70 used as a loading control; co-immunoprecipitation (*right panel*), with immunoisolation performed using anti-Sur1 antibody, and immunoblot performed using anti-Trpm4 antibody, showing co-assembly of Sur1 and Trpm4 exclusively in EAE. The results illustrated are representative of findings in five mice/group; scale bars, 100 μm (**a**–**d**); 200 μm (**e**); 50 μm (**f**)

Double immunolabeling experiments showed that, as with Sur1, Trpm4 was not detected in normal WT controls, exhibited modest immunopositivity in the white matter on pid-10, and showed robust labeling in white matter by pid-30 (Fig. 2a–c, e). Double labeling of tissues from *Abcc8*−/−/EAE mice showed upregulation of Trpm4 but no detectable Sur1 (Fig. 2d). As with Sur1, quantification of Trpm4 immunopositivity confirmed a progressive increase in white matter over the course of 30 days post-immunization (Fig. 2g).

The merged images of Fig. 2 showed striking co-localization of Trpm4 with Sur1, with quantitative analysis confirming a very high degree of co-localization (PCC, 0.94) (Fig. 1g). This finding suggested that astrocytes in WT/EAE mice might be expressing co-assembled Sur1-Trpm4 channels. To determine whether Sur1 co-assembled with Trpm4 to form Sur1-Trpm4 channels in EAE, we performed immunoFRET and co-immunoprecipitation, as previously described [17, 18]. ImmunoFRET imaging demonstrated that Sur1 and Trpm4 co-assembled (Fig. 2f). Measurements of FRET efficiency, when both primary antibodies were present, yielded values of 10–14 %, compared to ~0 % in negative controls [17, 18]. Co-immunoprecipitation experiments were carried out using tissues from pid-30 WT/EAE mice. These experiments showed that Sur1 was upregulated in EAE compared to controls (Fig. 2h). More importantly, co-assembled Sur1-Trpm4 was undetectable in controls but was prominent in EAE, confirming the findings of immunoFRET that Sur1 and Trpm4 co-assembled in EAE (Fig. 2h).

### Glibenclamide and *Abcc8*−/− in murine EAE

**Fig. 3:**
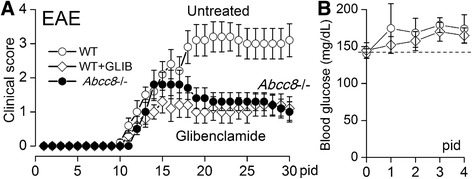
Glibenclamide and *Abcc8*−/− reduce disease severity in EAE. **a** Mean values of clinical scores following EAE induction for untreated WT/EAE mice, for glibenclamide-treated WT/EAE mice and *Abcc8*−/−/EAE mice; glibenclamide treatment was started on pid-10, when >20 % of mice had clinical scores of 1 or greater; 10 mice/group. **b** Daily serum glucose for first 4 days in mice receiving no treatment (*empty circle*) or glibenclamide treatment (empty diamonds)

Clinical scoring was assessed daily on pid-0–30. Untreated WT/EAE mice showed a typical course of acute EAE, marked by worsening paralysis scores beginning on pid-10–13, peaking several days later, then persisting through pid-30 (Fig. 3a). The mean clinical score for the disease period (pid-9–30) was 2.34 ± 0.23. The day of disease onset and the mean clinical scores at different times during the disease course are shown in Table 1.

**Table 1:** Summary of EAE clinical scores

Group	Disease incidence/total (%)	Number of deaths	Disease onset (days)^a^	Mean clinical scores				
				Day 15^b^	Day 20^c^	Day 25^c^	Day 30^c^	Day 9–30^d^
WT/EAE	10/10 (100)	2	11.8 ± 0.36	2.1 ± 0.38	3.1 ± 0.46	3.0 ± 0.45	3.1 ± 0.48	2.34 ± 0.23
WT/EAE + GLIB	10/10 (100)	1	12.9 ± 0.50	1.3 ± 0.34	1.0 ± 0.34	1.0 ± 0.33	1.1 ± 0.35	0.94 ± 0.08
*Abcc8*−/−/EAE	10/10 (100)	0	13.0 ± 0.41	1.8 ± 0.36	1.4 ± 0.31	1.3 ± 0.26	1.0 ± 0.29	1.16 ± 0.12

Values are presented as mean ± SEM (10 mice per group)

^a^Disease onset is defined as the first day of a clinical score of one or more. There was no statistically significant difference in day of onset of disease between the groups

^b^There was no statistically significant difference in scores between the groups on day 15

^c^On day 20, 25, and 30, the mean clinical scores of mice were significantly lower in WT/EAE + GLIB and *Abcc8*−/−/EAE groups compared to WT/EAE group. (For all three time points: WT/EAE vs. WT/EAE + GLIB, *P* < 0.01; WT/EAE vs. *Abcc8*−/−/EAE, *P* < 0.01; *N* = 10; one-way ANOVA with Fisher’s post-hoc comparisons)

^d^Overall Mean score during the disease phase (Day 9 to 30) is significantly lower in WT/EAE + GLIB and *Abcc8*−/−/EAE groups compared to WT/EAE group. (WT/EAE vs. WT/EAE + GLIB, *P* < 0.001; WT/EAE vs. *Abcc8*−/−/EAE, *P* < 0.001; *N* = 10; one-way ANOVA with Fisher’s post-hoc comparisons)

We used the potent Sur1 inhibitor, glibenclamide, to assess whether Sur1-Trpm4 channels play a role in disease progression. Mice were injected once daily with 10 μg IP, beginning at the time of disease onset (pid-10) and continuing through to pid-30. Compared to untreated WT/EAE mice, glibenclamide ameliorated the clinical manifestations of EAE (Fig. 3a). The decrease in clinical severity in glibenclamide-treated WT/EAE mice persisted throughout the experiment and manifested a robust effect size (Cohen’s *d*, 1.2). The mean clinical score for the disease period (pid-9–30) was 0.94 ± 0.08, which was significantly different from that in untreated WT/EAE mice (Table 1). Notably, the dose of glibenclamide used did not induce hypoglycemia (Fig. 3b).

The findings on Sur1-Trpm4 upregulation, combined with the effects observed with glibenclamide, were consistent with involvement of Sur1. To confirm functional involvement of Sur1 in EAE, we also studied *Abcc8*−/− mice. As with glibenclamide in WT/EAE mice, *Abcc8*−/−/EAE mice exhibited a decrease in clinical severity, compared to untreated WT/EAE mice, which persisted throughout the experiment and manifested the same robust effect size as glibenclamide (Fig. 3a). The mean clinical scores for the disease period (pid-9–30) was 1.16 ± 0.12, which was significantly different from untreated WT/EAE mice but not different from glibenclamide-treated WT/EAE mice (Table 1).

### Glibenclamide and *Abcc8*−/− reduce the inflammatory burden in EAE

**Fig. 4:**
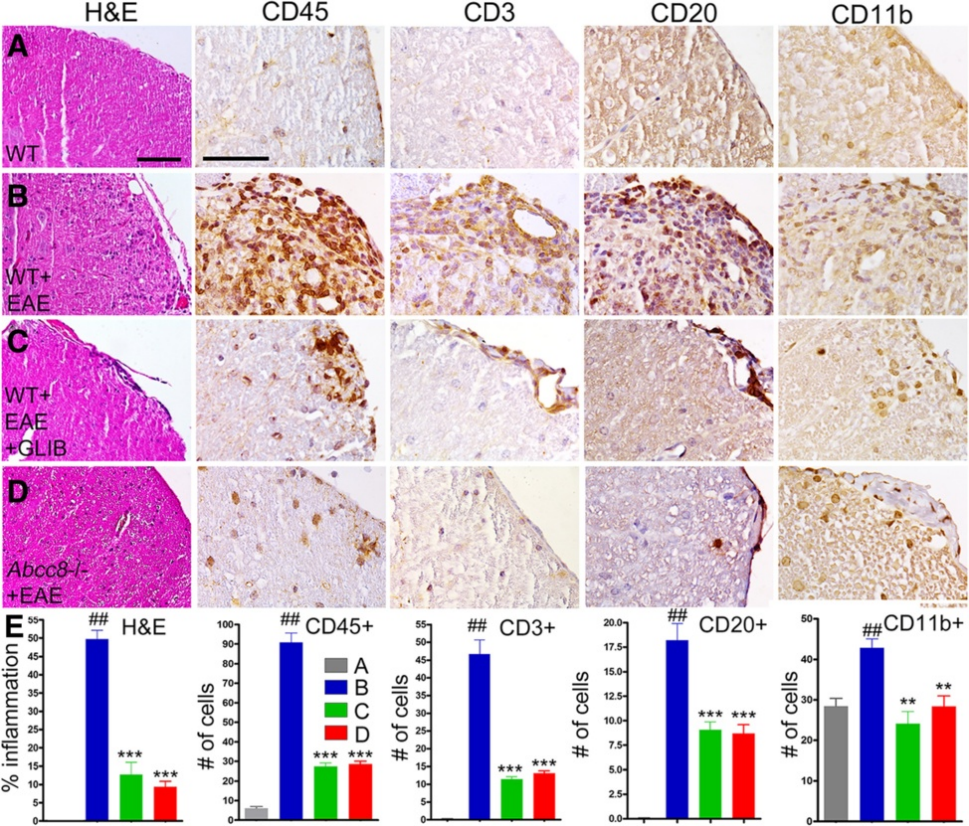
Glibenclamide and *Abcc8*−/− suppress immune cell infiltration in EAE. **a**–**d** White matter of lumbar spinal cord sections from WT control (**a**), untreated pid-30 WT/EAE (**b**), glibenclamide-treated pid-30 WT/EAE (**c**), and pid-30 *Abcc8*−/−/EAE (**d**) mice, stained with H&E or immunolabeled for CD45 (leucocyte), CD3 (T cells), CD20 (B cells), or CD11b (macrophage/microglia), as indicated; original magnification, ×200 (H&E) or ×400 (all immunolabelings). **e** *left panel*: percent of quadrants with inflammatory cells on H&E; four mice/group. **e** *four right panels*: Quantification of CD-45-, CD3-, CD20-, and CD11b-expressing cells in white matter; four mice/group; ^##^ *P* < 0.01 with respect to WT control; **P < 0.01, and ***P < 0.001 with respect to WT/EAE; scale bars, 100 μm

Sections of lumbar spinal cords from normal WT and *Abcc8*−/− controls (without EAE induction), untreated WT/EAE mice, glibenclamide-treated WT/EAE mice, and *Abcc8*−/−/EAE mice at pid-30 were stained with H&E (Fig. 4). Normal WT controls (Fig. 4a) and *Abcc8*−/− controls (not shown) were indistinguishable, showing no constitutive inflammation. Foci of inflammation were observed in the white matter of all untreated EAE spinal cords (Fig. 4b). Meningeal, perivascular, and parenchymal inflammatory infiltrates were reduced in the spinal cords of glibenclamide-treated WT/EAE and *Abcc8*−/−/EAE mice, compared to untreated WT/EAE mice (Fig. 4c, d). Quantification revealed a significant difference in the infiltrated cell accumulation, 49.7 ± 2.4 % of the quadrants in the untreated WT/EAE group were positive for inflammation, compared with 12.7 ± 3.3 % of the quadrants in glibenclamide-treated WT/EAE mice and 9.4 ± 1.5 % of the quadrants in *Abcc8*−/−/EAE mice (Fig. 4e).

To characterize the inflammatory cells present, sections of lumbar spinal cords were immunolabeled for markers of leukocytes (CD45), T cells (CD3), B cells (CD20), and macrophage/microglia (Cd11b). Inflammatory lesions in untreated WT/EAE lumbar spinal cords contained significantly increased numbers of CD45^+^, CD3^+^, CD20^+^, and CD11b^+^ cells, compared to normal controls (Fig. 4a, b, e). The number of inflammatory cells in glibenclamide-treated WT/EAE and *Abcc8*−/−/EAE lumbar spinal cords was significantly reduced, compared to untreated WT/EAE spinal cords (Fig. 4c–e).

**Fig. 5:**
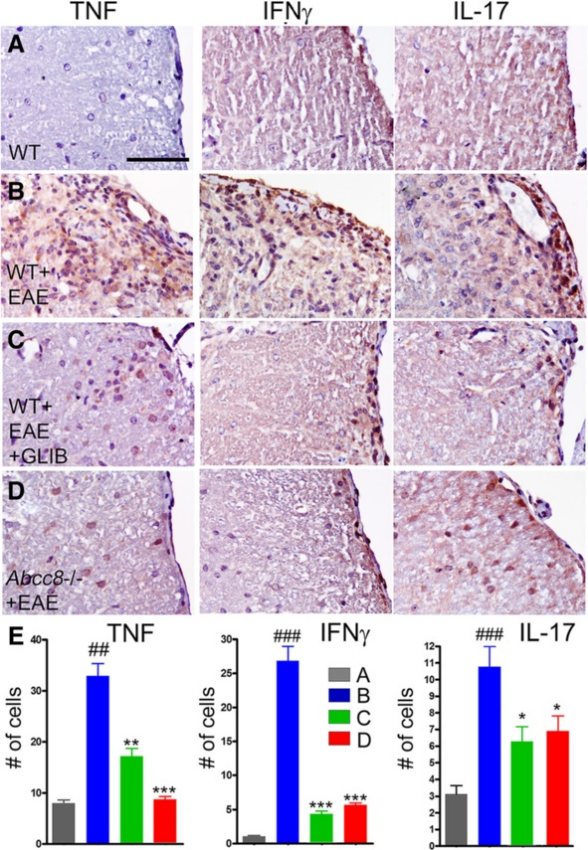
Glibenclamide and *Abcc8*−/− alter the cytokine profile in EAE. **a**–**d** White matter of lumbar spinal cord sections from WT control (**a**), untreated pid-30 WT/EAE (**b**), glibenclamide-treated pid-30 WT/EAE (**c**), and pid-30 *Abcc8*−/−/EAE (**d**) mice, immunolabeled for TNFα, IFN-γ, or IL-17, as indicated; original magnification, ×400. **e** Quantification of TNFα-, IFN-γ-, and IL-17-expressing cells in white matter; four mice/group; ^##^ *P* < 0.01 and ^###^ *P* < 0.001 with respect to WT control; *P < 0.05, **P < 0.01, and ***P < 0.001 with respect to WT/EAE; scale bars, 100 μm

To further characterize the effect of Sur1 inhibition or deletion on immune modulation in vivo, we counted cells positive for the pro-inflammatory cytokines TNF-α, IFN-γ, and IL-17, all of which were identified in white matter of lumbar spinal cord sections. Cells expressing TNF-α, IFN-γ, and IL-17 were significantly increased in untreated WT/EAE mice compared to normal controls (Fig. 5a, b, e). Cells expressing TNF-α, IFN-γ, and IL-17 were significantly decreased in glibenclamide-treated WT/EAE and *Abcc8*−/−/EAE mice, compared to untreated WT/EAE mice (Fig. 5c–e).

### Glibenclamide and *Abcc8*−/− reduce demyelination and promote remyelination in EAE

**Fig. 6:**
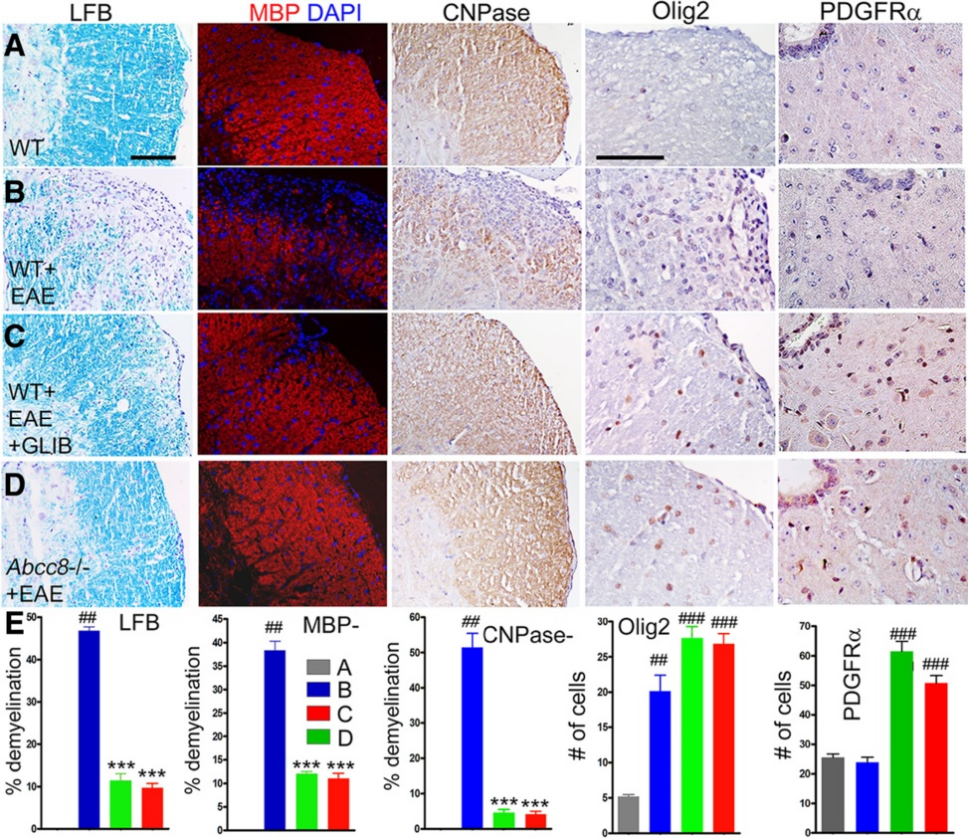
Glibenclamide and *Abcc8*−/− promote remyelination in EAE. **a**–**d** White matter of lumbar spinal cord sections from WT control (**a**), untreated pid-30 WT/EAE (**b**), glibenclamide-treated pid-30 WT/EAE (**c**), and pid-30 *Abcc8*−/−/EAE (**d**) mice, stained with Luxol fast blue (LFB) or immunolabeled for MBP (myelin), CNPase, Olig2 (oligodendrocytes), or PDGFR-α (oligodendrocyte progenitor cells (OPC)), as indicated; nuclei stained with DAPI in the MBP sections; original magnification, ×200 (LFB) or ×400 (all immunolabelings). **e** *three left panels*: Percent of quadrants with myelin loss by LFB staining, by MBP immunolabeling, or by CNPase immunolabeling. **e** *two right panels*: Quantification of Olig-2-expressing cells in white matter or of PDGFR-α-expressing OPC near the central canal; four mice/group; ^##^ *P* < 0.01 and ^###^ *P* < 0.001 with respect to WT control; ***P < 0.001 with respect to WT/EAE; scale bars, 100 μm

Spinal cord myelin was stained using Luxol fast blue (LFB) and was immunolabeled for myelin basic protein (MBP) [32] (Fig. 6). White matter tracts in untreated WT/EAE mice stained weakly with LFB and labeled weakly for MBP. Compared to normal controls, the spinal cords of untreated WT/EAE mice showed 46 ± 1 % demyelination by LFB and 38 ± 1.9 % by MBP, whereas the spinal cords of glibenclamide-treated WT/EAE mice showed 11 ± 2 % demyelination by LFB and 12.1 ± 0.5 % by MBP, and those of *Abcc8*−/−/EAE mice showed 9.7 ± 1.1 % demyelination by LFB and 11.1 ± 1.1 % by MBP.

Expression of the mature oligodendrocyte marker, CNPase, verified the data on MBP (Fig. 6). Compared to normal controls, 51 ± 0.4 % of the quadrants in untreated EAE mice showed loss of staining, compared to 4.5 ± 1.0 % of the quadrants in glibenclamide-treated WT/EAE mice and 4.2 ± 0.8 % of the quadrants in *Abcc8*−/−/EAE mice.

We also performed counts of cells positive for Olig-2, a marker of oligodendrocytes, and PDGFR-α, a marker of oligodendrocyte precursor cells (OPC) (Fig. 6). Olig-2^+^ but not PDGFR-α^+^ cells were significantly increased in WT/EAE, while cells with both markers were significantly increased in both glibenclamide-treated WT/EAE and in *Abcc8*−/−/EAE, consistent with increased proliferation of OPC with treatment.

### Glibenclamide and *Abcc8*−/− protect against axonal damage

**Fig. 7:**
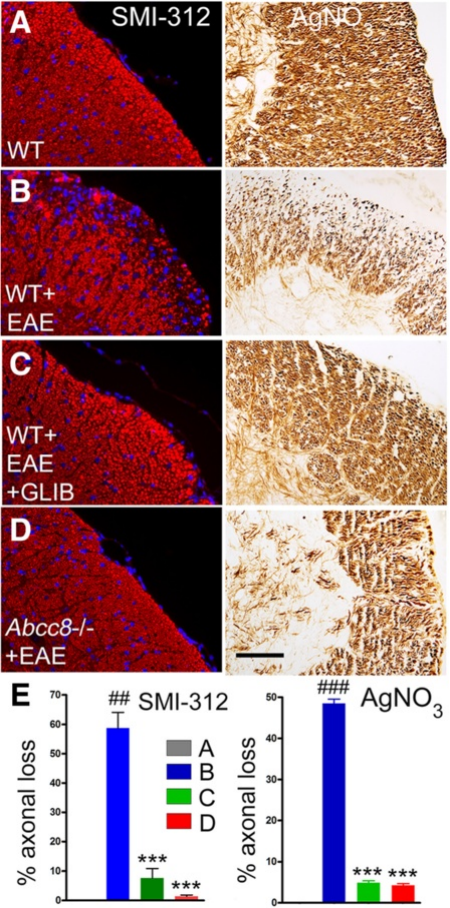
Glibenclamide and *Abcc8*−/− protect against axonal damage. **a**–**d** White matter of lumbar spinal cord sections from WT control (**a**), untreated pid-30 WT/EAE (**b**), glibenclamide-treated pid-30 WT/EAE (**c**), and pid-30 *Abcc8*−/−/EAE (**d**) mice, immunolabeled for SMI-312 (neurofilament marker) or stained with silver nitrate, as indicated; original magnification, ×400. **e** Percent of quadrants with axonal loss by SMI-132 labeling and by silver nitrate staining; four mice/group; ^##^ *P* < 0.01 and ^###^ *P* < 0.001 with respect to WT control; ***P < 0.001 with respect to WT/EAE; scale bars, 100 μm

To assess for axonal sparing, spinal cords were examined using the pan-axonal neurofilament marker, anti-SMI 312, as well as silver nitrate staining (Fig. 7). For SMI 312 and silver nitrate, respectively, quantification revealed axonal losses of 58.7 ± 5.3 % and 48.5 ± 1 % in untreated WT/EAE mice, compared to normal controls, versus losses of 7.7 ± 3.2 % and 4.9 ± 0.5 % in glibenclamide-treated WT/EAE mice and 1.4 ± 0.4 % and 4.3 ± 0.4 % in *Abcc8*−/−/EAE mice.

## Discussion

The major findings of the present study are that in EAE (i) Sur1 and Trpm4 are progressively upregulated between pid-10 and pid-30, (ii) Sur1 and Trpm4 co-assemble to form Sur1-Trpm4 channels, (iii) the dominant cell type that expresses Sur1-Trpm4 is the astrocyte, and (iv) deletion as well as pharmacological blockade of Sur1 yields robust neurological protection in EAE.

The clinical course and severity of EAE were significantly ameliorated in WT/EAE mice administered glibenclamide beginning at the time of disease onset (pid-10) as well as in *Abcc8*−/−/EAE mice. On pid-30, the lumbar spinal cords of WT/EAE mice treated with glibenclamide and of *Abcc8*−/−/EAE mice showed a significantly reduced inflammatory burden, including fewer inflammatory lesions (H&E), fewer invading peripheral immune cells, including leukocytes (CD45), T cells (CD3), B cells (CD20) and macrophages/microglia (CD11b), and fewer cells expressing pro-inflammatory cytokines (TNF-α, IFN-γ, IL-17). The reduced inflammatory burden with glibenclamide and *Abcc8* deletion correlated with better preservation of myelin (LFB, MBP), better preservation of axons (silver nitrate, SMI-312), and more numerous mature and precursor oligodendrocytes (CNPase, Olig-2, PDGFR-α). Glibenclamide and *Abcc8* deletion also increased the density of CNPase as well as MBP, which are markers of mature OLs in vivo. The improved myelination with glibenclamide and *Abcc8* deletion may have resulted from an enhanced number of OPCs differentiating into myelinating OLs, as these treatments increased the numbers and promoted the maturation of myelinating cells.

Schattling et al. [20] were the first to report the effect of glibenclamide in a murine MOG_35–55_ model of EAE. In their report, Schattling et al. attributed the beneficial effects of glibenclamide to blockade of Trpm4. However, the present study casts doubt on their interpretation that Trpm4 is the *direct* target of glibenclamide in EAE. First, we show here that Trpm4 upregulation is accompanied by upregulation of Sur1 and by co-assembly of Trpm4 with Sur1 to form Sur1-Trpm4 heteromers. It is known that glibenclamide is much more potent as a blocker of Sur1-Trpm4 than of Trpm4 alone—the EC_50_ for glibenclamide blockade of Sur1-Trpm4 is 48 nM, and both native and recombinant Sur1-Trpm4 channels are blocked >90 % by 1 μM [17, 33]. By contrast, with Trpm4 alone, the EC_50_ for glibenclamide may be as high as 100 μM [34], and 1 μM results in <10 % blockade [17]. The dose of glibenclamide administered by Schattling et al., as well as by us in the present report, was 10 μg per mouse, ~0.4 mg/kg, daily. In rodents, this dose yields peak serum levels of ~120 nM [35, 36], which is far below that required to block Trpm4 alone but is adequate for blockade of Sur1-Trpm4. Second, the observations that (i) protection by *Abcc8* deletion is indistinguishable from protection by glibenclamide and (ii) in *Abcc8−/−* mice, Trpm4 was upregulated yet appeared to be harmless in the absence of Sur1, not only confirmed functional involvement of Sur1 in EAE but also is most consistent with the hypothesis that protection with glibenclamide is due to Sur1 inhibition, not Trpm4 inhibition.

Apart from blockade of Sur1-regulated channels, glibenclamide exhibits other actions that could potentially contribute to the salutary effects observed here and previously [20]. Glibenclamide is known to block the NLRP3 (NACHT, LRR, and PYD domains-containing protein 3) inflammasome, which has been implicated in the pathophysiology of EAE [37]. However, given the high dose of glibenclamide required to block the inflammasome (EC_50_, ~75 μM) [38], it is unlikely that this mechanism was involved in the beneficial effect of glibenclamide in EAE. Glibenclamide also acts as a PPARγ (peroxisome proliferator-activated receptor γ) agonist [39], a class of drugs with favorable effects in CNS inflammation, including EAE [40, 41]. However, glibenclamide’s efficacy as a PPARγ agonist is only ~20 % that of pioglitazone [39]. Importantly, since neither the NLRP3 inflammasome nor PPARγ involves Sur1, and since deletion of *Abcc8* mimicked the effect of glibenclamide, the involvement of either of these mechanisms is highly unlikely. Overall, our data indicate that Sur1-Trpm4 is the most likely target of glibenclamide in EAE.

An important property of the Sur1-Trpm4 channel is that both subunits, Sur1 and Trpm4, are required for the manifestation of its pathological effects. This pathognomonic property previously was best exemplified in traumatic spinal cord injury, where pharmacological blockade of Sur1 (glibenclamide, repaglinide) or of Trpm4 (flufenamic acid, riluzole), gene suppression (antisense oligodeoxynucleotide against *Abcc8* or *Trpm4*), and gene deletion (*Abcc8−/−* or *Trpm4*−/−), all were shown to result in the exactly the same phenotype—reduced microvascular dysfunction and capillary fragmentation [24]. Our present data, combined with those of Schattling et al. [20], extend observations based on traumatic spinal cord injury, showing that in EAE as well, deletion of *Abcc8* or of *Trpm4* results in the same phenotype—reduced inflammation and better preservation of myelin, better preservation of axons, and more numerous mature and precursor oligodendrocytes.

Schattling et al. [20] showed that immune cell infiltration, measured on pid-15, was not affected by glibenclamide treatment beginning on pid-8. By contrast, we found a significant reduction in immune cell infiltrates with glibenclamide when tissues were studied on pid-30. Apart from being performed much earlier, the cell counts reported by Schattling et al. [20] were based on the entire CNS (brain and spinal cord), whereas our cell counts were based on the lumbar spinal cord alone, where the pathological manifestations of EAE are the greatest [31]. It could be that, in the Schattling study, counting invading immune cells in the larger volume of tissue, most of which likely was unaffected at pid-15, inadvertently masked elevated numbers in the lumbar spinal cord.

Schattling et al. [20] reported significantly more Trpm4-positive axons in EAE mice compared to controls, based on morphology showing labeling of small round structures. Surprisingly, Schattling et al. did not report Trpm4 expression in astrocytes, whereas our data showed robust expression of Sur1-Trpm4 in astrocytes in EAE. These apparent differences are almost certainly due to the different times at which tissues were evaluated after disease induction. Schattling et al. studied tissues on pid-14—our data showed minimal expression on pid-10 but robust expression by pid-30. It is not surprising that astrocytes would express Sur1 and Trpm4, since this is the cell type in which the Sur1-Trpm4 channel was first discovered [33] and in which it has been repeatedly shown to be upregulated post-injury [17, 18, 42]. Notably, in cerebral ischemia, expression by astrocytes also increases slowly, reaching a maximum only after 1 month [29].

The prominent expression of Sur1-Trpm4 by astrocytes suggests that astrocytic Sur1-Trpm4 channels may be a principal target of glibenclamide. There is emerging recognition of a critical role of astrocytes as immune effector cells with an essential role in EAE [43–46]. Activation of astrocytes in EAE occurs at the onset of the acute clinical episode, with the intensity being a good predictor of the clinical severity in animal models [47]. Astrocytes are the first cells in the CNS to be activated by MOG-reactive T cells and to synthesize pro-inflammatory cytokines and chemokines that are essential for the induction of EAE [48]. As active players in CNS innate immunity, astrocytes participate actively and differently at different stages of the pathologic process [43]. Astrocytes contribute mechanistically to lesion development in EAE by (i) modifying blood–brain barrier properties and up-regulating adhesion molecules and matrix metalloproteases required for leukocyte invasion, (ii) expressing cytokines and chemokines that attract leukocytes, (iii) producing factors toxic to oligodendrocytes and neurons, and (iv) blocking the maturation of oligodendrocyte precursor cells.

Important questions remain about Sur1-Trpm4 in EAE. Although the Sur1-Trpm4 channel in astrocytes has been characterized [33, 49], the role of the channel in astrocyte function in EAE remains to be determined. It is known that the channel acts principally as a negative regulator of calcium influx, with block of the channel by glibenclamide promoting increased calcium influx [17, 50]. Astrocytes secrete pro-inflammatory cytokines and chemokines that are essential for the induction of EAE [48, 51]. It may be that Sur1-Trpm4 blockade by glibenclamide alters calcium signaling in astrocytes and thereby impairs their secretion of pro-inflammatory cytokines and chemokines. Additional work, including astrocyte-specific deletion of Sur1-Trpm4 in EAE, will be required to fully elucidate the role of astrocytic Sur1-Trpm4 channels in the pathogenesis of EAE.

## Conclusions

The Sur-Trpm4 channel is newly upregulated in murine EAE. Given its prominent expression in reactive astrocytes, and the important pro-inflammatory role of astrocytes in MS and EAE, the Sur1-Trpm4 channel may represent a novel therapeutic approach for disease modification to reduce peripheral immune cell entry into the CNS without compromising the function of peripheral immune cells themselves.

## References

[1] Dutta R, Trapp BD (2011). Mechanisms of neuronal dysfunction and degeneration in multiple sclerosis. Prog Neurobiol.

[2] International Multiple Sclerosis Genetics C, Wellcome Trust Case Control C, Sawcer S, Hellenthal G, Pirinen M, Spencer CC (2011). Genetic risk and a primary role for cell-mediated immune mechanisms in multiple sclerosis. Nature.

[3] Mc Guire C, Volckaert T, Wolke U, Sze M, de Rycke R, Waisman A (2010). Oligodendrocyte-specific FADD deletion protects mice from autoimmune-mediated demyelination. J Immunol.

[4] Hohlfeld R (1997). Biotechnological agents for the immunotherapy of multiple sclerosis. Principles, problems and perspectives. Brain.

[5] Peterson LK, Fujinami RS (2007). Inflammation, demyelination, neurodegeneration and neuroprotection in the pathogenesis of multiple sclerosis. J Neuroimmunol.

[6] Becher B, Bechmann I, Greter M (2006). Antigen presentation in autoimmunity and CNS inflammation: how T lymphocytes recognize the brain. J Mol Med.

[7] Gold R, Linington C, Lassmann H (2006). Understanding pathogenesis and therapy of multiple sclerosis via animal models: 70 years of merits and culprits in experimental autoimmune encephalomyelitis research. Brain.

[8] Sospedra M, Martin R (2005). Immunology of multiple sclerosis. Annu Rev Immunol.

[9] Krakowski ML, Owens T (2000). Naive T lymphocytes traffic to inflamed central nervous system, but require antigen recognition for activation. Eur J Immunol.

[10] Wingerchuk DM, Carter JL (2014). Multiple sclerosis: current and emerging disease-modifying therapies and treatment strategies. Mayo Clin Proc.

[11] Colombo E, Di Dario M, Capitolo E, Chaabane L, Newcombe J, Martino G (2014). Fingolimod may support neuroprotection via blockade of astrocyte nitric oxide. Ann Neurol.

[12] Choi JW, Gardell SE, Herr DR, Rivera R, Lee CW, Noguchi K (2011). FTY720 (fingolimod) efficacy in an animal model of multiple sclerosis requires astrocyte sphingosine 1-phosphate receptor 1 (S1P1) modulation. Proc Natl Acad Sci U S A.

[13] Becerra A, Echeverria C, Varela D, Sarmiento D, Armisen R, Nunez-Villena F (2011). Transient receptor potential melastatin 4 inhibition prevents lipopolysaccharide-induced endothelial cell death. Cardiovasc Res.

[14] Simard JM, Woo SK, Gerzanich V (2012). Transient receptor potential melastatin 4 and cell death. Pflugers Archiv.

[15] Gerzanich V, Woo SK, Vennekens R, Tsymbalyuk O, Ivanova S, Ivanov A (2009). De novo expression of Trpm4 initiates secondary hemorrhage in spinal cord injury. Nat Med.

[16] Loh KP, Ng G, Yu CY, Fhu CK, Yu D, Vennekens R (2014). TRPM4 inhibition promotes angiogenesis after ischemic stroke. Pflugers Archiv.

[17] Woo SK, Kwon MS, Ivanov A, Gerzanich V, Simard JM (2013). The sulfonylurea receptor 1 (Sur1)-transient receptor potential melastatin 4 (Trpm4) channel. J Biol Chem.

[18] Tosun C, Kurland DB, Mehta R, Castellani RJ, De Jong JL, Kwon MS (2013). Inhibition of the Sur1-Trpm4 channel reduces neuroinflammation and cognitive impairment in subarachnoid hemorrhage. Stroke.

[19] Mehta RI, Tosun C, Ivanova S, Tsymbalyuk N, Famakin BM, Kwon MS (2015). Sur1-Trpm4 cation channel expression in human cerebral infarcts. J Neuropathol Exp Neurol.

[20] Schattling B, Steinbach K, Thies E, Kruse M, Menigoz A, Ufer F (2012). TRPM4 cation channel mediates axonal and neuronal degeneration in experimental autoimmune encephalomyelitis and multiple sclerosis. Nat Med.

[21] Hollenstein K, Dawson RJ, Locher KP (2007). Structure and mechanism of ABC transporter proteins. Curr Opin Struct Biol.

[22] Aittoniemi J, Fotinou C, Craig TJ, de Wet H, Proks P, Ashcroft FM (2009). Review. SUR1: a unique ATP-binding cassette protein that functions as an ion channel regulator. Philos Trans R Soc Lond B Biol Sci.

[23] Shi NQ, Ye B, Makielski JC (2005). Function and distribution of the SUR isoforms and splice variants. J Mol Cell Cardiol.

[24] Simard JM, Woo SK, Aarabi B, Gerzanich V. The Sur1-Trpm4 channel in spinal cord injury. Journal of spine. 2013;Suppl 4. doi:10.4172/2165-7939.S4-002. 10.4172/2165-7939.S4-002 PMC4019017 24834370

[25] Seghers V, Nakazaki M, DeMayo F, Aguilar-Bryan L, Bryan J (2000). Sur1 knockout mice. A model for K(ATP) channel-independent regulation of insulin secretion. J Biol Chem.

[26] Makar TK, Bever CT, Singh IS, Royal W, Sahu SN, Sura TP (2009). Brain-derived neurotrophic factor gene delivery in an animal model of multiple sclerosis using bone marrow stem cells as a vehicle. J Neuroimmunol.

[27] Makar TK, Trisler D, Bever CT, Goolsby JE, Sura KT, Balasubramanian S (2008). Stem cell based delivery of IFN-beta reduces relapses in experimental autoimmune encephalomyelitis. J Neuroimmunol.

[28] Dunn KW, Kamocka MM, McDonald JH (2011). A practical guide to evaluating colocalization in biological microscopy. Am J Physiol Cell Physiol.

[29] Mehta RI, Ivanova S, Tosun C, Castellani RJ, Gerzanich V, Simard JM (2013). Sulfonylurea receptor 1 expression in human cerebral infarcts. J Neuropathol Exp Neurol.

[30] Nimmagadda VK, Bever CT, Vattikunta NR, Talat S, Ahmad V, Nagalla NK (2013). Overexpression of SIRT1 protein in neurons protects against experimental autoimmune encephalomyelitis through activation of multiple SIRT1 targets. J Immunol.

[31] Arima Y, Harada M, Kamimura D, Park JH, Kawano F, Yull FE (2012). Regional neural activation defines a gateway for autoreactive T cells to cross the blood-brain barrier. Cell.

[32] Barbarese E, Barry C, Chou CH, Goldstein DJ, Nakos GA, Hyde-DeRuyscher R (1988). Expression and localization of myelin basic protein in oligodendrocytes and transfected fibroblasts. J Neurochem.

[33] Chen M, Dong Y, Simard JM (2003). Functional coupling between sulfonylurea receptor type 1 and a nonselective cation channel in reactive astrocytes from adult rat brain. J Neurosci.

[34] Demion M, Bois P, Launay P, Guinamard R (2007). TRPM4, a Ca2 + −activated nonselective cation channel in mouse sino-atrial node cells. Cardiovasc Res.

[35] Chakradhar L, Kallem R, Karthik A, Sundari BT, Ramesh S, Mullangi R (2008). A rapid and highly sensitive method for the determination of glimepiride in human plasma by liquid chromatography-electrospray ionization tandem mass spectrometry: Application to a pre-clinical pharmacokinetic study. Biomed Chromatogr.

[36] Ao Y, Chen J, Yue J, Peng RX (2008). Effects of 18alpha-glycyrrhizin on the pharmacodynamics and pharmacokinetics of glibenclamide in alloxan-induced diabetic rats. Eur J Pharmacol.

[37] Inoue M, Shinohara ML (2013). NLRP3 inflammasome and MS/EAE. Autoimmune Dis.

[38] Lamkanfi M, Mueller JL, Vitari AC, Misaghi S, Fedorova A, Deshayes K (2009). Glyburide inhibits the Cryopyrin/Nalp3 inflammasome. J Cell Biol.

[39] Fukuen S, Iwaki M, Yasui A, Makishima M, Matsuda M, Shimomura I (2005). Sulfonylurea agents exhibit peroxisome proliferator-activated receptor gamma agonistic activity. J Biol Chem.

[40] Bright JJ, Kanakasabai S, Chearwae W, Chakraborty S (2008). PPAR regulation of inflammatory signaling in CNS diseases. PPAR Res.

[41] Drew PD, Xu J, Racke MK (2008). PPAR-gamma: therapeutic potential for multiple sclerosis. PPAR Res.

[42] Simard JM, Chen M, Tarasov KV, Bhatta S, Ivanova S, Melnitchenko L (2006). Newly expressed SUR1-regulated NC(Ca-ATP) channel mediates cerebral edema after ischemic stroke. Nat Med.

[43] Brosnan CF, Raine CS (2013). The astrocyte in multiple sclerosis revisited. Glia.

[44] Guo X, Nakamura K, Kohyama K, Harada C, Behanna HA, Watterson DM (2007). Inhibition of glial cell activation ameliorates the severity of experimental autoimmune encephalomyelitis. Neurosci Res.

[45] Jensen CJ, Massie A, De KJ (2013). Immune players in the CNS: the astrocyte. J Neuroimmune Pharmacol.

[46] Nair A, Frederick TJ, Miller SD (2008). Astrocytes in multiple sclerosis: a product of their environment. Cell MolLife Sci.

[47] Luo J, Ho P, Steinman L, Wyss-Coray T (2008). Bioluminescence in vivo imaging of autoimmune encephalomyelitis predicts disease. J Neuroinflammation.

[48] Kang Z, Altuntas CZ, Gulen MF, Liu C, Giltiay N, Qin H (2010). Astrocyte-restricted ablation of interleukin-17-induced Act1-mediated signaling ameliorates autoimmune encephalomyelitis. Immunity.

[49] Chen M, Simard JM (2001). Cell swelling and a nonselective cation channel regulated by internal Ca2+ and ATP in native reactive astrocytes from adult rat brain. J Neurosci.

[50] Simard JM, Woo SK, Schwartzbauer GT, Gerzanich V (2012). Sulfonylurea receptor 1 in central nervous system injury: a focused review. J Cereb Blood Flow Metab.

[51] Paul D, Ge S, Lemire Y, Jellison ER, Serwanski DR, Ruddle NH (2014). Cell-selective knockout and 3D confocal image analysis reveals separate roles for astrocyte-and endothelial-derived CCL2 in neuroinflammation. J Neuroinflammation.

